# Soy milk versus simvastatin for preventing atherosclerosis and left ventricle remodeling in LDL receptor knockout mice

**DOI:** 10.1590/1414-431X20165854

**Published:** 2017-02-20

**Authors:** L. Santos, A.P. Davel, T.I.R. Almeida, M.R. Almeida, E.A. Soares, G.J.M. Fernandes, S.F. Magalhães, V.G. Barauna, J.A.D. Garcia

**Affiliations:** 1 Unidade Acadêmica de Serra Talhada, Universidade Federal Rural de Pernambuco, Serra Talhada, PE, Brasil; 2 Departamento de Biologia Estrutural e Funcional, Instituto de Biologia, Universidade de Campinas, Campinas, SP, Brasil; 3 Instituto Federal do Sul de Minas, Muzambinho, MG, Brasil; 4 Departamento de Anatomia, Instituto de Ciências Biomédicas, Universidade Federal de Alfenas, Alfenas, MG, Brasil; 5 Departmento de Biomedicina, Universidade José do Rosário Vellano, Alfenas, MG, Brasil; 6 Departamento de Ciências Fisiológicas, Universidade Federal do Espírito Santo, Vitória, ES, Brasil; 7 Departmento de Tecnologia, Ciência e Educação, Instituto Federal do Sul de Minas, Machado, MG, Brasil; 8 Departamento de Fisiologia, Universidade José do Rosário Vellano, Alfenas, MG, Brasil

**Keywords:** Soy milk, Dyslipidemia, Oxidative stress, Inflammation, Atherosclerosis, Simvastatin

## Abstract

Functional food intake has been highlighted as a strategy for the prevention of cardiovascular diseases by reducing risk factors. In this study, we compared the effects of oral treatment with soy milk and simvastatin on dyslipidemia, left ventricle remodeling and atherosclerotic lesion of LDL receptor knockout mice (LDLr-/-) fed a hyperlipidic diet. Forty 3-month old male LDLr-/- mice were distributed into four groups: control group (C), in which animals received standard diet; HL group, in which animals were fed a hyperlipidic diet; HL+SM or HL+S groups, in which animals were submitted to a hyperlipidic diet plus soy milk or simvastatin, respectively. After 60 days, both soy milk and simvastatin treatment prevented dyslipidemia, atherosclerotic lesion progression and left ventricle hypertrophy in LDLr-/- mice. These beneficial effects of soy milk and simvastatin were associated with reduced oxidative stress and inflammatory state in the heart and aorta caused by the hyperlipidic diet. Treatment with soy milk was more effective in preventing HDLc reduction and triacylglycerol and VLDLc increase. On the other hand, simvastatin was more effective in preventing an increase in total cholesterol, LDLc and superoxide production in aorta, as well as CD40L both in aorta and left ventricle of LDLr-/-. In conclusion, our results suggest a cardioprotective effect of soy milk in LDLr-/- mice comparable to the well-known effects of simvastatin.

## Introduction

Dyslipidemia is a major risk factor for cardiovascular diseases (CVD) such as coronary heart disease, cerebrovascular disease and peripheral arterial disease, which are still the world's leading cause of death (1). Serum low density lipoprotein cholesterol (LDLc)-lowering drugs have been reported to decrease CVD morbimortality through interruption, reversion or prevention of vascular injury and left ventricle hypertrophy (LVH) associated with primary and secondary dyslipidemias (2,3).

Oxidative stress has been associated with dyslipidemia, and several studies suggest an important relationship between cardiovascular oxidative stress, inflammation, atherosclerotic disease, and LVH (2,4). Excessive vascular reactive oxygen species (ROS) generation could mediate cellular damage, necrosis and apoptosis via DNA, protein and lipid oxidation (5), besides provoking endothelial dysfunction and inflammatory cells infiltration and activation (6). Vascular inflammation has a crucial role in the pathogenesis of atherosclerosis, accelerating atheroma plaque formation, progression, and plaque destabilization and rupture, which precedes CVD clinical outcomes (4). Accordingly, upregulation of CD40 receptor and its ligand CD40L in vascular tissue and left ventricle (LV) are found during all stages of atherosclerosis (7) and LVH associated with dyslipidemia (2).

Reduction in LDLc by hydroxymethylglutaryl coenzyme A (HMG CoA) reductase inhibitors (statins) remain the most clinically validated therapy to reduce cardiovascular events by reducing plasma LDL, very low (VL)DL, inflammatory markers and increasing nitric oxide (NO) (8). However, long-term statins treatment is associated to skeletal muscle complaints including myositis, rhabdomyolysis, serum creatine kinase increase, myalgia and muscular weakness. It has been reported the association of statins with several conditions, such as renal and hepatic compromise, hypothyroidism, diabetes, polyneuropathy, and other side effects (9).

Isoflavones (also referred as isoflavonoids) are a class of phytoestrogens widely distributed in the vegetable kingdom. The concentrations of these chemical phenolic compounds are relatively high in legumes. In soy bean (*Glycine max*), daidzein, genistein and glycitein and derivatives are the main isoflavones, which are presented in various forms of glycoside conjugates in accordance to the processing extension or fermentation (10). Drozen and Harrison (11) defined functional foods as food products that provided specific health benefits beyond the traditional nutrients they contain. Functional foods as therapy for controlling dyslipidemias are an important strategy since phytoestrogens ingestion has a high impact over hypercholesterolemia [total cholesterol (TC) and LDLc], showing level A of evidence (multiple randomized controlled clinical trials) (8).

Beneficial effects of soy derivative products are associated with HDLc metabolism and reduction in LDLc in some populations (12). However, the results are still inconclusive and outcomes range from confirmed benefits, to few benefits, to no benefits (12). Therefore, the aim of the present study was to compare the effects of soy milk on dyslipidemia, cardiac remodeling and atherosclerotic injury in LDL receptor knockout (LDLr-/-) mice fed a hyperlipidic diet to the well-known protective effects of simvastatin.

## Material and Methods

### Animals

The experiments were performed in homozygotic 3-month-old male LDLr deficient mice (LDLr-/-) generated in the background C57BL6/J, weighing 22±3 g. The mice were acquired from Jackson Laboratory (USA) and bred in the Bioterio da Universidade José do Rosário Vellano (UNIFENAS, Alfenas, MG, Brazil, with controlled temperature and 12/12-h light-dark cycle environment. LDLr-/- mice (n=10/group) were fed with a standard diet (Nuvital, Brazil) as the control group (C), or with a hyperlipidic diet (HL group; 20% total fat, 1.25% cholesterol and 0.5% cholic acid), with water and food *ad libitum*. HL-fed animals received water (vehicle), soy milk (HL+SM group; 0.6 mL soy milk AdeS Original¯, Unilever, Brazil), or simvastatin (HL+S group; 20 mg·kg^-1^·day^-1^, Medley, Brazil) daily *per gavage* for 60 days. Experimental procedures were performed in accordance with the guidelines established by the National Council for Animal Experiments Control (CONCEA) and were approved by the Animals Ethics Committee of the Instituto Federal do Sul de Minas Gerais (No. 03A/2014).

Blood samples were obtained by retro-orbital venous plexus puncture in anesthetized animals (xylazine/ketamine, 6/40 mg/kg, respectively; Bayer AS/Parke-Davis, USA) for glucose, insulin, triacylglycerol (TG), TC and LDLc, high density lipoprotein cholesterol (HDLc), VLDLc fractions serum analyses. Subsequently, after thoracotomy, heart and aorta were perfused *in situ* with 1.34 mM KCl followed by 10% PBS-buffered formaldehyde. The heart was removed and LV was isolated. LV weight (mg)/body weight (g) ratio was used as LVH index. LV and thoracic aorta were fixed for 24 h in 10% formaldehyde.

### Serum analysis

Serum was obtained by centrifugation (1200 *g*, 4^o^C, 10 min). Glucose serum level was measured by colorimetric enzymatic method. Insulin serum level was determined using a commercial specific ELISA kit (DAKO Ltda., UK). Homa index (Homa-ir) was calculated by the formula: {Homa-ir = [fast insulinemia (mU/L) × fast glycemia (mmol/L)] / 22.5} to determine insulin resistance. Enzymatic trials were used for measuring TG, TC and HDLc, as described by Hedrick et al. (13). C-reactive protein (CRP) level was calculated by turbidimetry and photometry (Humastar 300¯, Human Diagnostics, Germany) and results are reported in mg/dL (14).

### LDLc and VLDLc determination

LDLc was determined according to Friedewald et al. (15): LDLc (mg/dL) = TC-HDLc – TG/5.0, and VLDLc according to Tian et al. (16): VLDLc (mg/dL) = TG/5.0

### Superoxide anion measurement in aorta

Superoxide anion levels were assessed on thoracic aortic homogenates by using lucigenin (5 μmol/L) chemiluminescence, as described by Laurindo et al. (17). Results are reported as counts per minute (cpm)/mg protein, quantified by Bradford method.

### Histological analysis

The hearts were embedded sequentially in 5, 10, and 25% gelatin. Oil red staining was carried out in aortic root according to Paigen et al. (18) and blind-quantified as previously described (19) using Image Pro Plus software (version 3.0) for image analysis (Media Cybernetics, USA).

For morphometric analyses of cardiomyocytes diameter and collagen deposition, 4-µm width sections from LV embedded in paraffin were stained with hematoxylin-eosin and picrosirius red, respectively. Photomicrographs were taken from the same prefixed point of LV cross sections of each mouse using a digital camera coupled to the Leica IM50 (Germany). Cardiomyocyte diameter was measured from 8 to 12 cells in each section per animal (20). Picrosirius red stained sections were submitted to a polarized light and each photomicrograph was analyzed by the LGMC-image software (Lafayette General Medical Center, USA) (21).

### Immunohistochemistry

LV and abdominal aorta histological sections were treated with 3% hydrogen peroxide to block endogenous peroxidase activity. Unspecific sites blockage was performed with 2% skimmed milk diluted in phosphate-buffered saline (10 mM PBS, pH=7.4). Glass slides containing tissue sections were incubated for 12 h in a humid chamber at 4°C with polyclonal antibody (1:50; Santa Cruz, USA) anti-CD40L. After, sections were washed with PBS and incubated with a biotinylated secondary antibody (Dako LSAB) in a humid chamber for 1 h at 37°C. In order to visualize immunoreactive areas, glass slides were incubated with the conjugate complex with peroxidase (Dako LSAB) for 45 min at 37°C and put in a chromogenic solution (50 mg DAB in 50 mL PBS with 3 mL 10% oxygen water) for 3 min. After counterstaining with Harris hematoxylin (Sigma¯, USA) for 25 s, glass slides were mounted and analyzed by optical microscopy. Photomicrographs were analyzed by the LGMC-image software (2).

### Statistical analyses

Data are reported as means±SE. One-way analyses of variance (ANOVA) followed by Tukey’s test was used to compare means among different groups. Differences were considered to be significant at P<0.05.

## Results

Table 1 reports the serum biochemistry profile of each group. HL group exhibited severe dyslipidemia compared with C group, represented by increased serum TG and TC values. In addition, HL group exhibited marked decrease in HDLc fraction and marked increase in LDLc and VLDLc fractions compared with C group, as well as increased CRP. Soy milk and simvastatin treatment prevented the increase in TG, TC, LDLc and VLDc, as well as the decrease in HDLc. However, soy milk was more effective than simvastatin in preventing the increase in serum levels of TG and VLDLc and the decrease in HDLc caused by the hyperlipidic diet. There was no difference in serum glucose among groups. HL group showed hyperinsulinemia compared with C group, with increased Homa-ir_._ These changes were partially prevented in HL+SM group while completely prevented in HL+S group. There was no significant difference in body weight among groups (C=23.0±2.0 g; HL=23.3±1.8 g; HL+SM=23.5±1.9 g; HL+S=23.6±1.7 g).

**Table:**
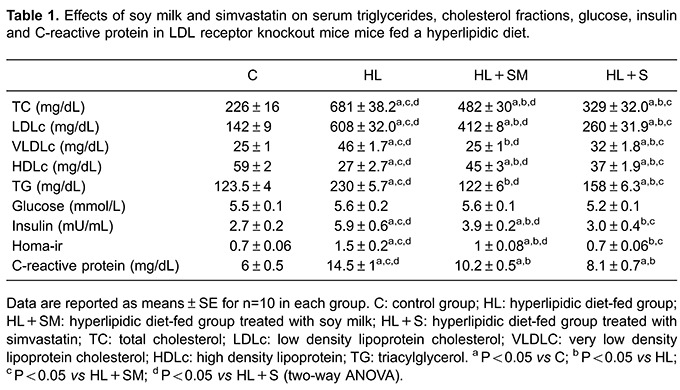

**Figure 1:**
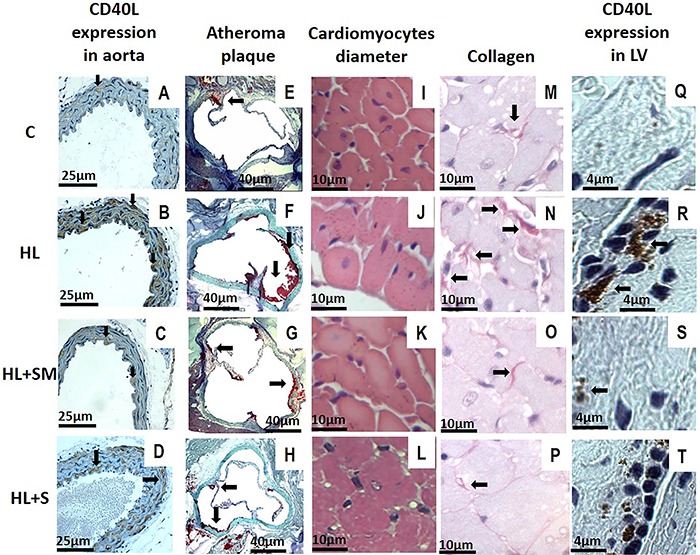
Representative photomicrographs of aortic section with immunohistochemical peroxidase staining to CD40L (A–D) and aortic atherosclerotic plaques (E–H). Representative photomicrographs of left ventricle sections showing cardiomyocytes diameter (I–L), collagen content (M–P) and immunohistochemical peroxidase staining to CD40L (Q–T). C: control group; HL: hyperlipidic diet-fed group; HL+SM: hyperlipidic diet-fed group treated with soy milk; HL+S: hyperlipidic diet-fed group treated with simvastatin.

Aorta from HL group exhibited increased superoxide anion level compared with C group. In addition, aorta of HL group also exhibited greater CD40L-immunoreactivity and plaque area compared with C group aortas. Soy milk and simvastatin treatment reduced these alterations in aorta tissue. However, simvastatin was more effective than soy milk in preventing the increased CD40L-immunoreactivity and superoxide anion level (Table 2 and Figure 1).

**Table:**
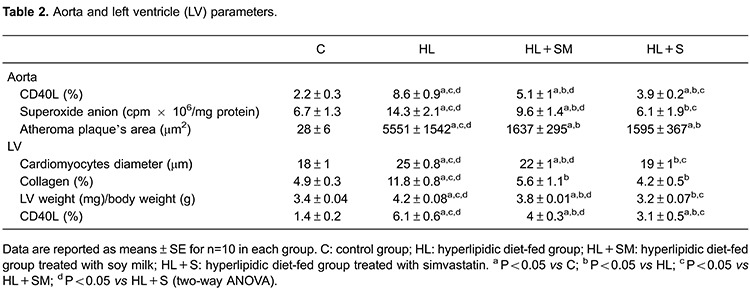

HL group developed cardiac hypertrophy, indicated by a 38% increase in cardiomyocytes diameter and 24% in the LV weight/body weight ratio. Also, HL group presented fibrosis, indicated by a 140% increase in collagen fraction compared with C group. Although simvastatin was more effective in preventing LV remodeling (hypertrophy and fibrosis) and CD40L immunoreactivity, treatment with soy milk was also effective in preventing LV remodeling and inflammation induced by the hyperlipidic diet in LDLr-/- mice (Table 2 and Figure 1).

## Discussion

In the present study, oral daily treatment with soy milk showed a beneficial effect in preventing the expression of pro-inflammatory factors CD40L and CRP, and oxidative stress in aorta and LV, as well as in improving severe mixed dyslipidemia and insulin resistance in LDLr-/- mice. These mechanisms were associated with a partial prevention of atherosclerotic lesion and LV remodeling induced by the hyperlipidic diet, and were comparable to the pharmacological therapy with simvastatin. Soy milk was more effective in preventing HDLc serum level decrease, and TG and VLDLc increase, while simvastatin was more effective in preventing TC and LDLc serum level increase, inflammation, ROS production and LV remodeling.

LDLr-/- mice develop spontaneous moderate hyperlipidemia, but are resistant to the development of neointimal lesions in carotid arteries (16), and show moderate oxidative stress (19) and lower insulin secretion (22) when fed with standard diet. However, high-fat diet induces neointimal lesion (16), hyperinsulinemia (23), vascular oxidative stress and LV remodeling (2) in LDLr-/- mice. Therefore, LDLr-/- mice submitted to a hyperlipidic diet represent an interesting model to study strategies to prevent atherosclerosis development.

In this study, the severe dyslipidemia observed in hyperlipidic diet-fed LDLr-/- mice was characterized by a marked increase in TG, TC and LDLc and VLDLc fractions, as well as reduction in HDLc. Also, these animals showed elevated serum levels of insulin. A previous study has demonstrated increased TC and insulin secretion in LDLr-/- mice fed with high-fat diet, associated with hyperglycemia and impaired glucose tolerance (23). Interestingly, in the present study, glycemia was not different among groups. Different composition in the high-fat diet used by previous studies (23) may explain this discrepancy.

Superoxide anion production increased in aorta of HL group suggesting that hyperlipidic diet aggravates the moderate oxidative stress in LDLr-/- mice fed with standard diet (19). In addition, LDLr-/- mice fed a hyperlipidic diet showed upregulation of CD40L-immunoreactive areas in both aorta and LV as well as increased serum C-reactive protein. Oxidative stress and enhanced inflammatory markers are associated with increased atherosclerotic lesion and LV remodeling. Accordingly, previous studies have demonstrated increased superoxide anion formation in aorta and cardiac tissue from LDLr-/- mice fed hyperlipidic diet (19). In addition, it has been shown that a hyperlipidic diet increases left ventricle hypertrophy, CD40L staining, and collagen amount (3). CD40L is an important inflammatory signaling molecule involved in different stages of atherosclerosis development, from the beginning to acute complications after atheroma plaque’s rupture (24).

Previous studies had demonstrated the antioxidant role of soy and its biological active forms daidzein, genistein and glycitein. Kanazawa et al. (25) observed LDL, VLDL and HDL lipid peroxidation decrease in patients that received soy cream after a stroke event. In line with this, individuals consuming three soy bars a day for two weeks exhibited decreased susceptibility to LDLc oxidation compared with non-consumers of soy bars (26). Kapiotis et al. (27) demonstrated that the soy biologic form genistein inhibits LDLc oxidation in the presence of copper ions or superoxide radicals *in vitro*. Besides that, the study suggested that genistein was effective in protecting endothelial cells from damage caused by oxidized lipoproteins. In agreement with these previous studies, we observed that soy milk was effective in reducing oxidative stress induced by hyperlipidic diet in aorta from LDLr-/- mice and, importantly, atheroma plaque area was three times smaller in LDLr^-/-^ mice treated with soy milk than in the non-treated group.

Studies have shown that the hypocholestorolemic effect of soy is in part due to isoflavones (28). The soy milk used here has an isoflavones concentration of 11.7 mg/200 mL (29). Isoflavones or isoflavonoids pertain to polyphenols group, which has antioxidant properties. However, isoflavones show a chemical structure similar to that of estrogens and are commonly referred as phytoestrogens. The possible effects by which phytoestrogens reduced cholesterol in the group HL+SM are related to the modulation of intestinal cholesterol absorption and may: i) restrict cholesterol solubility in micelles, which decreases food lipid emulsification and consequently hampers lipase action and TG hydrolysis into free fatty acids monoacylglycerol and diacylglycerol (8,30); ii) compete with cholesterol for Niemann-Pick C1-like 1 protein, part of an intestinal cholesterol transport system that is located in enterocytes' apical membrane and promotes cholesterol passage through the brush border, thus decreasing intestinal absorption (31); and iii) reduce cholesterol esterification and chylomicrons synthesis within intestinal enterocytes, decreasing chylomicrons lymphatic absorption (32). In 2016, Dong et al. (33) showed that the consumption of soy milk with phytosterols also decreased serum TC and LDLc levels in older Chinese people from both ApoE3 and ApoE4 genotypes. This study strengthens the knowledge about the effects of soy milk on other models of dyslipidemia.

Also, HL+SM mice showed decreased LV and aortic levels of inflammatory markers such as CD40L and C-reactive protein. This anti-inflammatory effect of soy milk was associated with a marked increase in serum levels of HDLc. As HDL was found to have anti-inflammatory and antioxidant properties (34), these could be mechanisms involved in the beneficial metabolic and cardiovascular effect of soy milk treatment. Some studies have demonstrated that HDLc represses adhesion molecules transcription, such as CD11b/CD18, Selectin E, VCAM 1 and ICAM 1, to decrease cytokines and chemo-cytokines production, such as TNF-α, IL-6, IL-10 and MCP-1, and to inhibit AP-1 and NFκB activation (35). Thus, beneficial effects of soy milk may be due to the association of direct isoflavone actions and the indirect HDLc increase, reducing oxidative stress and inflammatory response.

Lastly, simvastatin also prevented lipid profile changes, hyperinsulinemia, LVH and atheroma plaque area in the HL group. These metabolic and cardiovascular effects of simvastatin may be associated with antioxidant and anti-inflammatory response in LV and aorta, as demonstrated by a reduced CD40L immunoreactivity area, reduced serum CRP, as well as reduced superoxide anion production. By reducing mevalonate and isoprenyl radical formation, statins attenuate the activation of inflammatory molecules such as Intercellular Adhesion Molecule 1 (ICAM-1), NF-κB e CD40L (36). Statins also reduce ROS generation by vascular NADPH-oxidase and have been reported to have a direct anti-inflammatory effect eliminating free radicals, thereby contributing to increased synthesis of the vasodilatory and anti-thrombotic factor NO (37).

In conclusion, soy milk showed important beneficial metabolic and cardiovascular effects preventing severe dyslipidemia, hyperinsulinemia, atherosclerotic lesion, LVH and collagen deposition in LDLr-/- mice fed a hyperlipidic diet. These protective effects were quantitatively comparable to the effects of simvastatin and were associated with an improved redox balance and reduction of systemic and cardiovascular inflammatory factors (CD40L and CPR). Thus, our study contributes to the scientific literature by emphasizing the potential role of soy derivative products to prevent dyslipidemia, atherogenesis, cardiovascular remodeling and insulin resistance.
